# Microsecond melting and revitrification of cryo samples

**DOI:** 10.1063/4.0000129

**Published:** 2021-10-26

**Authors:** Jonathan M. Voss, Oliver F. Harder, Pavel K. Olshin, Marcel Drabbels, Ulrich J. Lorenz

**Affiliations:** Laboratory of Molecular Nanodynamics, École Polytechnique Fédérale de Lausanne, 1015 Lausanne, Switzerland

## Abstract

The dynamics of proteins that are associated with their function typically occur on the microsecond timescale, orders of magnitude faster than the time resolution of cryo-electron microscopy. We have recently introduced a novel approach to time-resolved cryo-electron microscopy that affords microsecond time resolution. It involves melting a cryo sample with a heating laser, so as to allow dynamics of the proteins to briefly occur in the liquid phase. When the laser is turned off, the sample rapidly revitrifies, trapping the particles in their transient configurations. Precise control of the temperature evolution of the sample is crucial for such an approach to succeed. Here, we provide a detailed characterization of the heat transfer occurring under laser irradiation as well as the associated phase behavior of the cryo sample. While areas close to the laser focus undergo melting and revitrification, surrounding regions crystallize. *In situ* observations of these phase changes therefore provide a convenient approach for assessing the temperature reached in each melting and revitrification experiment and for adjusting the heating laser power on the fly.

## INTRODUCTION

Proteins enable living systems to process energy, self-regulate, respond to stimuli, grow, and reproduce. As nanoscale machines, they are inherently dynamic systems that undergo conformational rearrangements as they perform their tasks.^1–4^ The characteristic timescales involved typically range from microseconds to milliseconds.^5,6^ While cryo-electron microscopy (cryo-EM)^7–10^ is rapidly becoming the preferred method in structural biology,^11^ its time resolution is currently too low to observe such fast dynamics, leaving our understanding of protein function fundamentally incomplete.^6^ In time-resolved cryo-EM, dynamics are usually induced by rapidly mixing two reactants or exposing the sample to a short stimulus, such as a pulse of light, after which the sample is rapidly plunge-frozen to trap the particles in short-lived states.^12–16^ However, the time required for plunge-freezing limits the time resolution of this technique to several milliseconds,^16,17^ too slow to observe many relevant processes.

We have recently demonstrated a novel approach to time-resolved cryo-EM with microsecond time resolution, three orders of magnitude faster than previously possible.^18,19^ Our method involves melting a cryo sample with a laser beam for a duration of tens of microseconds or longer, which provides a tunable time window during which a stimulus can cause the particles to undergo conformational dynamics. When the heating laser is switched off, the sample revitrifies within just a few microseconds,^18^ trapping the particles in their transient configurations, which can be subsequently imaged with the electron beam. A short laser pulse can serve as a suitable stimulus to induce dynamics, either by directly triggering a photoactive protein or by releasing a caged compound, such as ions, ATP, or peptides.^20,21^ Some types of stimuli, such as the release of caged compounds, may already be applied before the sample is melted. Since the particles are unable to undergo conformational dynamics while they are trapped in the vitreous ice matrix, the stimulus only becomes active once the sample is melted.

## RESULTS

**FIG. 1.**
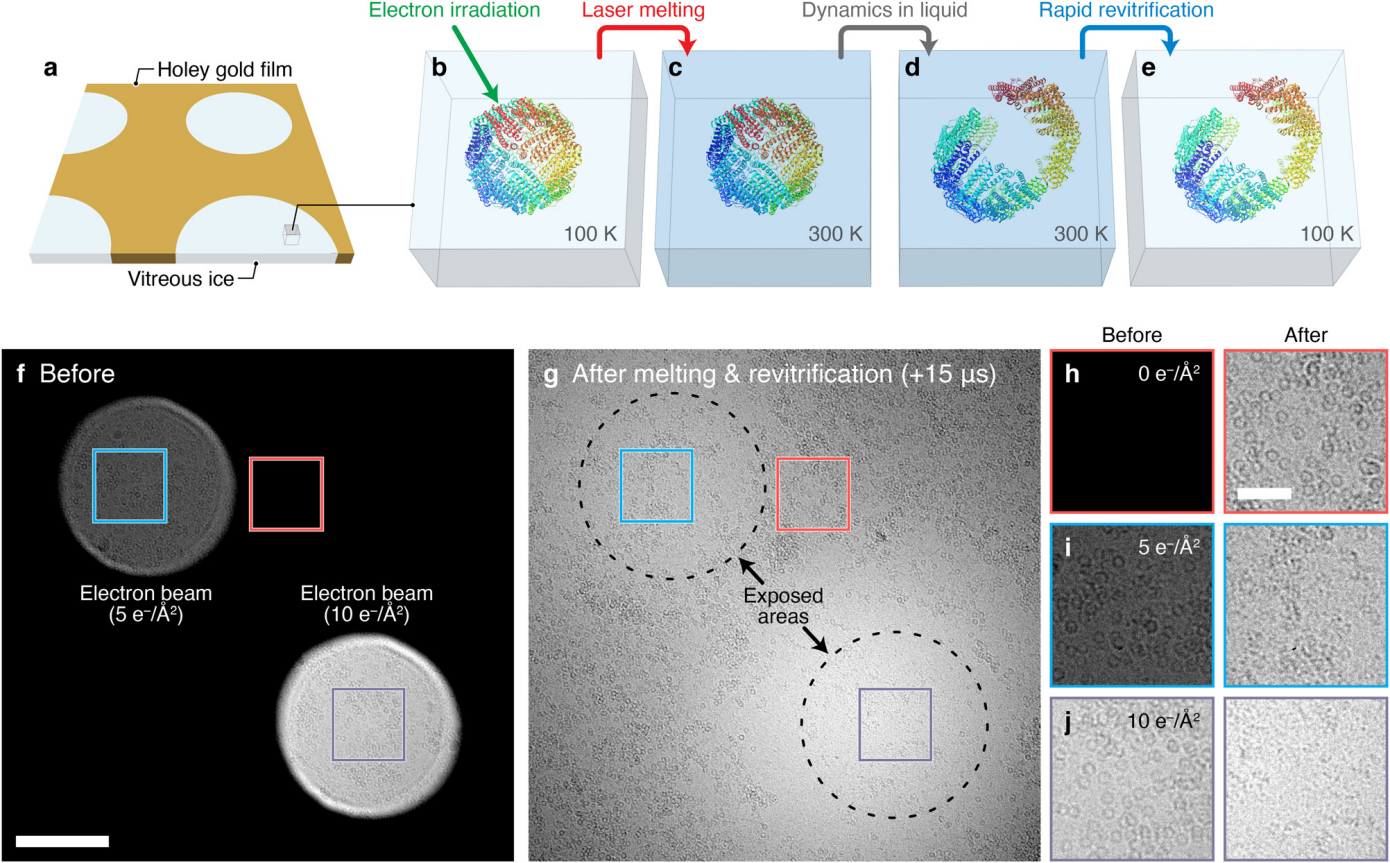
Rapid melting and revitrification of cryo samples, experimental concept, and demonstration. (a) Illustration of the geometry of the cryo sample, which is supported by a holey gold film. (b)–(e) Experimental concept. (b) Apoferritin particles are irradiated with the electron beam, which alters their structure and thus serves as a stimulus for dynamics to occur. (c) The cryo sample is melted *in situ* by heating with a laser, (d) allowing the structure of the particles to freely evolve. (e) Once the laser is switched off, the sample rapidly revitrifies and particles are arrested in their transient configurations, which can be subsequently imaged with conventional cryo-EM techniques. Cartoon of apoferritin adapted from Ref. 24. (f)–(j) Proof-of-principle demonstration. (f) Composite micrograph of a cryo sample of apoferritin in which only the two circular areas in the top left and bottom right have been exposed with a dose of 5 and 10 electrons/Å^2^, respectively. Scale bar, 200 nm. (g) The sample is melted *in situ* with a 15 *μ*s laser pulse and revitrifies. Particles that were illuminated with the electron beam prior to melting have unraveled during the short period when the sample was liquid, while those in the unexposed areas remain intact. (h)–(j) Details of the square areas marked in (f) and (g). Scale bar, 50 nm.

Figure 1 presents a demonstration of such a melting and revitrification experiment that makes use of the well-established phenomenon that particles incur electron beam damage during cryo-imaging.^22,23^ Vitreous ice is believed to fix fragments in place, thereby preserving the structure of the particles during imaging.^22,23^ In contrast, melting the sample should allow fragments to diffuse apart. In our experiment, we study a cryo sample of apoferritin on a holey gold film^24^ [Fig. 1(a)]. The sample is irradiated with the electron beam, which induces fragmentation and thus acts as a stimulus for structural changes to occur [Fig. 1(b)]. Laser melting the sample [Fig. 1(c)] should then allow the damaged particles to unravel in liquid [Fig. 1(d)] and permit us to trap them in partially disassembled configurations when the heating laser is switched off and the sample rapidly revitrifies [Fig. 1(e)]. The experiment confirms this expectation. In the composite micrograph of Fig. 1(f), we have illuminated the sample only in the top left and bottom right with a dose of 5 and 10 electrons/Å^2^, respectively, thus damaging only the proteins in these areas. We subsequently melt and revitrify the sample *in situ* with a 15 *μ*s laser pulse^25^ (532 nm, 63 mW, 24 ± 1 *μ*m spot size, see supplementary material Note S1 and Fig. S1),^46^ after which we record the micrograph in Fig. 1(g). The regions previously exposed to the electron beam (dashed circles) have visibly thinned, likely due to the evaporation of radiolysis products^23^ that were liberated when the ice was melted. The particles in these areas have disassembled during the short time when the sample was liquid, leaving only fragments that offer little contrast. As evident in the details of the areas marked with squares, disassembly is more extensive at higher electron dose [Figs. 1(i) and 1(j)]. In contrast, particles in the unexposed parts remain intact [Fig. 1(h)].

**FIG. 2.**
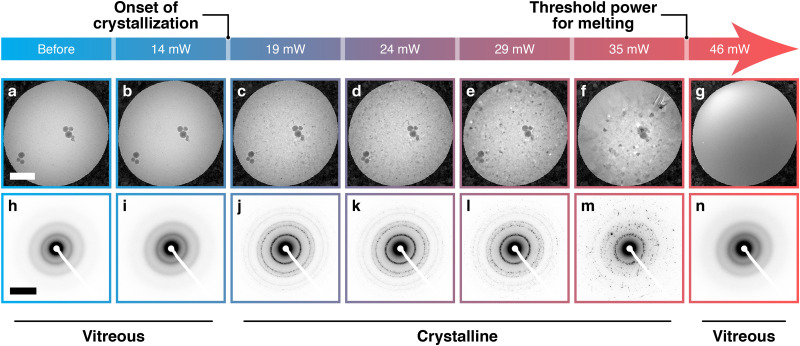
Phase behavior of cryo samples heated with laser pulses of increasing power. (a)–(g) Micrographs of a cryo sample under exposure to laser pulses of increasing power. (a) The sample (b) remains vitreous after heating with a 10 *μ*s laser pulse of 14 mW power, but (c) crystallizes at a power of 19 mW. (d)–(f) The crystal morphology changes as the power is increased in steps to 35 mW. (g) A single pulse of 46 mW power melts the sample, causing it to revitrify when it cools after the end of the laser pulse. (h)–(m) Diffraction patterns of a second identical cryo sample under exposure to laser pulses of the same powers as in (a)–(f). (n) Diffraction pattern of the revitrified sample in (g). Scale bars, 500 nm and 5 nm^−1^.

The success of melting and revitrification experiments crucially depends on the ability to accurately adjust the laser power, so that the sample reaches the desired temperature during the laser pulse. As shown in Fig. 2, we calibrate the laser power *in situ* by heating a cryo sample with laser pulses of increasing power until it reaches the melting point of ice. The micrograph in Fig. 2(a) shows a cryo sample on a holey gold film, where the hole that is visible is located in the center of a square of the gold mesh supporting the sample. Figure 2(b) shows that irradiation with a 10 *μ*s laser pulse of 14 mW power leaves the sample unchanged. Here and in the following experiments, the laser beam is centered on the area under observation. In contrast, a 19 mW laser pulse induces devitrification [Fig. 2(c)], as is observed when a cryo sample is warmed above a temperature of 150 K (Ref. 26). When we heat the sample with pulses of increasing power, the ice crystals can be seen to grow [Figs. 2(d)–2(f)]. Finally, at a laser power of 46 mW, the sample melts and revitrifies [Figs. 2(g) and 2(n)], allowing us to identify the power at which the sample reaches the melting point of ice.^18^ To heat the sample to a specific temperature exceeding the melting point, the laser power can be adjusted by comparison with heat transfer simulations, as discussed below.

Figures 2(h)–2(m) show diffraction patterns of a second identical cryo sample, recorded after heating it with pulses of the same laser powers as above (see also Fig. S2). The diffraction patterns confirm that the sample transforms from vitreous ice [Fig. 2(i)] to a mixture of cubic and hexagonal ice [Figs. 2(j)–2(m)]. Finally, Fig. 2(n) displays a diffraction pattern of the revitrified sample in Fig. 2(g), in which diffraction spots originating from ice crystals are notably absent.

**FIG. 3.**
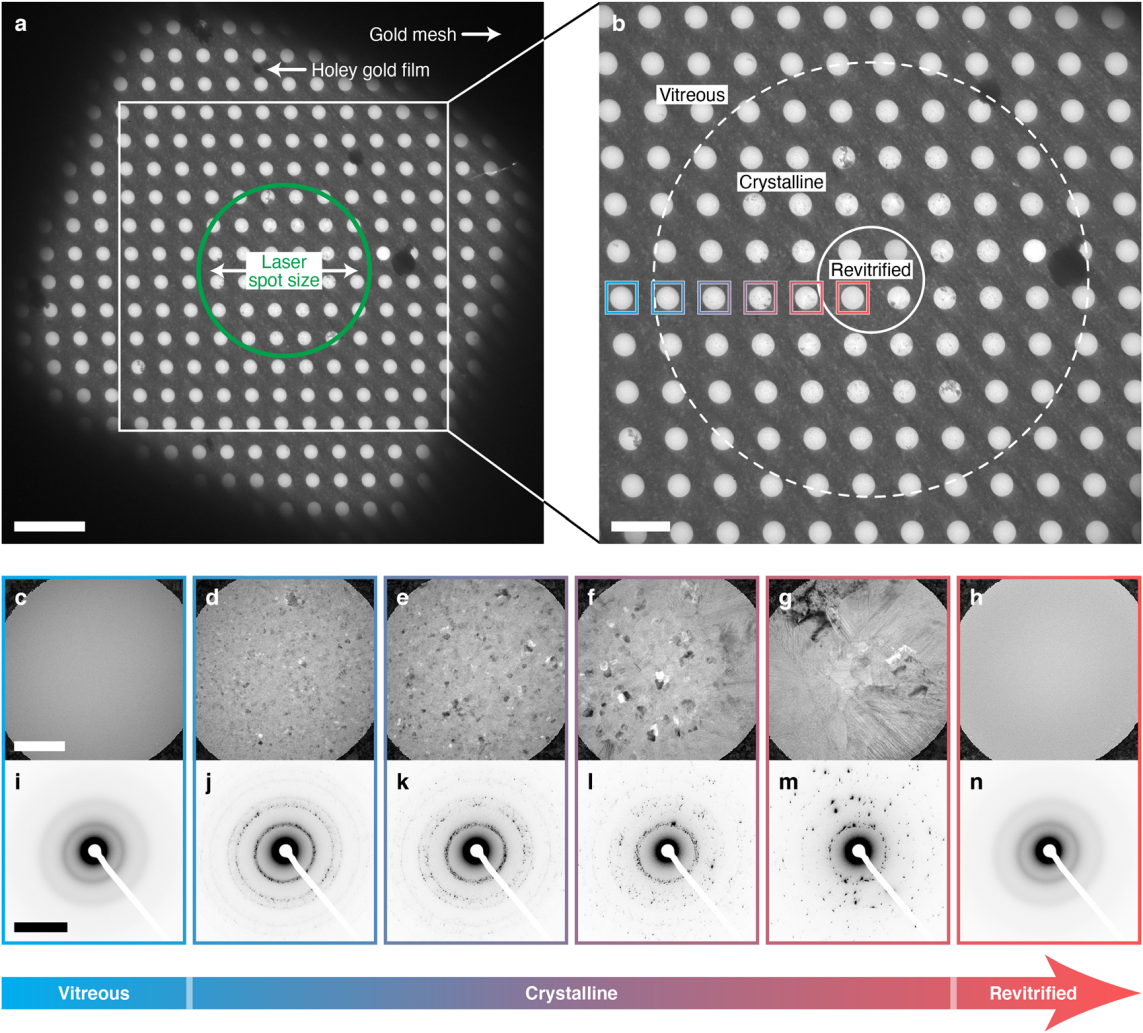
Phase behavior of a cryo sample in a melting and revitrification experiment. (a) Micrograph of a cryo sample after irradiation with a 10 *μ*s laser pulse (46 mW). The green circle indicates the laser position and spot size (24 *μ*m FWHM). Scale bar, 10 *μ*m. (b) Enlarged view of the region marked with the white square in (a). Melting and revitrification have occurred in all but one of the holes in the area marked with the small solid circle, while the sample has crystallized within the bounds of the large dashed circle. The rest of the sample has remained vitreous. Scale bar, 5 *μ*m. (c)–(h) Micrographs and (i)–(n) diffraction patterns of the regions marked with colored squares in (b). Scale bars, 500 nm and 5 nm^−1^.

As illustrated in Fig. 3, the phase behavior of the cryo sample under laser irradiation also provides a convenient means to verify *in situ* that the sample has reached the desired temperature in an individual melting and revitrification experiment as well as to make adjustments of the laser power on the fly. Figure 3(a) shows a low magnification micrograph of a cryo sample after irradiation with a 10 *μ*s laser pulse (46 mW). As above, the laser beam has been centered onto the square of the gold mesh. The laser spot size is indicated with a green circle. In a time-resolved cryo-EM experiment, the laser power is typically adjusted to quickly heat the sample to room temperature. Here, we have instead chosen a laser power that will just barely heat the sample above the melting point in the center of the laser focus, as established with the procedure detailed in Fig. 2. As discussed in the following, this choice facilitates the interpretation of our experiments. An enlarged view of the area marked with a square in Fig. 3(a) reveals a spatial variation of the phase behavior of the sample [Fig. 3(b)]. As desired for a time-resolved cryo-EM experiment, the sample has melted and revitrified in the center of the laser focus (small solid circle). In contrast, crystallization is apparent in the surrounding areas (large dashed circle), suggesting that further from the laser focus, where the sample has not been heated above the melting point, it has devitrified. At even larger distances closer to the grid bars, the sample has remained vitreous, having apparently not exceeded the crystallization temperature. The spatial variation of the phase behavior is also evident in micrographs [Figs. 3(c)–3(h)] and diffraction patterns [Figs. 3(i)–3(n)] of the areas marked with colored squares in Fig. 3(b). Near the center of the laser focus and close to the bars of the gold mesh, the homogeneous contrast of the micrographs [Figs. 3(c) and 3(h)] and diffuse rings of the diffraction patterns [Figs. 3(i) and 3(n)] confirm the presence of vitreous ice. In contrast, the areas in between exhibit crystals, which increase in size closer to the laser focus [Figs. 3(d)–3(g)] and whose diffraction patterns identify mixtures of cubic and hexagonal ice [Figs. 3(j)–3(m)].

**FIG. 4.**
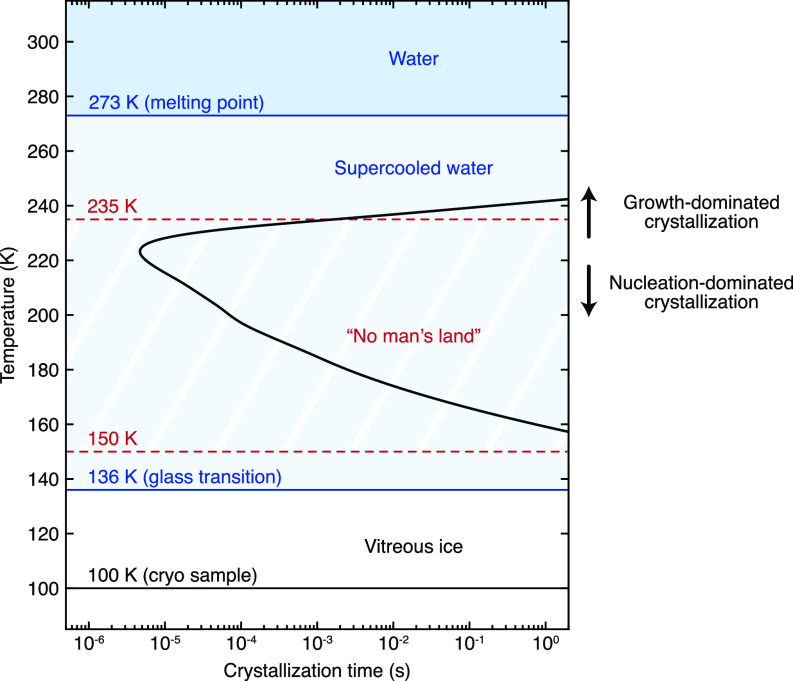
Time–temperature–transformation diagram of supercooled water. The black curve indicates the crystallization time of supercooled water, which is estimated from experimental nucleation^43^ and growth^44^ rates (see Note S2). It exhibits a minimum of 5 *μ*s at about 225 K, in a region of the phase diagram of water commonly referred to as “no man's land,”^30–34^ where rapid crystallization hinders the characterization of supercooled water. Crystallization is dominated by rapid crystal growth above 225 K, while nucleation dominates at lower temperatures.^35^ The solid blue lines indicate transition temperatures,^27^ while the dashed red lines denote the boundaries of “no man's land.”^26,45^

The phase behavior of the cryo sample as well as the crystal morphology in the devitrified areas can be understood when considering a time–temperature–transformation diagram of supercooled water, as shown in Fig. 4. Irradiation of the sample (∼100 K) with a laser pulse rapidly heats the vitreous ice above its glass transition temperature (136 K), so that it melts into a supercooled liquid.^27^ As the temperature rises further, the crystallization time of this metastable liquid decreases dramatically. The estimated crystallization time^28,29^ (black curve, see also Note S2 and Fig. S3) reaches a minimum of about 5 *μ*s around 225 K. Due to the rapid crystallization between 150 and 235 K, this temperature range is frequently referred to as “no man's land,” where the characterization of supercooled water has largely remained elusive.^30–34^ Rapid crystallization in “no man's land” notably causes the formation of cubic ice in the cryo sample of Fig. 2(c) when it is heated with a microsecond laser pulse of less than half the power needed to reach the melting point. We note that the crystallization times are estimated for pure water samples and therefore provide only a qualitative indication of the actual crystallization rates in our cryo samples.

In the experiment of Fig. 3, the laser power was adjusted such that the sample reaches the melting point only at the center of the laser focus. Correspondingly, we observe that only a small region of about 9-*μ*m-diameter revitrifies. Heat transfer simulations confirm that its size closely matches that of the area whose temperature exceeds the melting point by the end of the laser pulse, as discussed in more detail below. The crystallization of the surrounding areas likely does not occur while the sample cools, since the cooling rate of >10^7^ K/s is much higher than is needed to outrun crystallization, as previously shown,^18^ which allows the sample to revitrify in the center of the laser focus. Instead, it appears that crystallization already occurs during laser heating, as the sample traverses “no man's land”. Only those areas that are subsequently heated above the melting point will then become liquid again and revitrify after the heating laser is switched off.

The interpretation that crystal formation occurs during laser heating is also consistent with the spatial variation of the size of the crystallites that we observe in the crystalline regions [Figs. 3(d)–3(g)]. Below 225 K, the crystallization process is characterized by fast nucleation, but slow crystal growth. The opposite holds above this temperature, where nucleation slows dramatically, and crystallization is dominated by fast crystal growth (Fig. S3).^35^ Sample areas closer to the laser focus heat up more rapidly and therefore spend less time at temperatures below 225 K, where nucleation rates are high. A smaller number of nuclei is thus formed that can subsequently grow to a larger size once the temperature exceeds 225 K, in agreement with our observations.

**FIG. 5.**
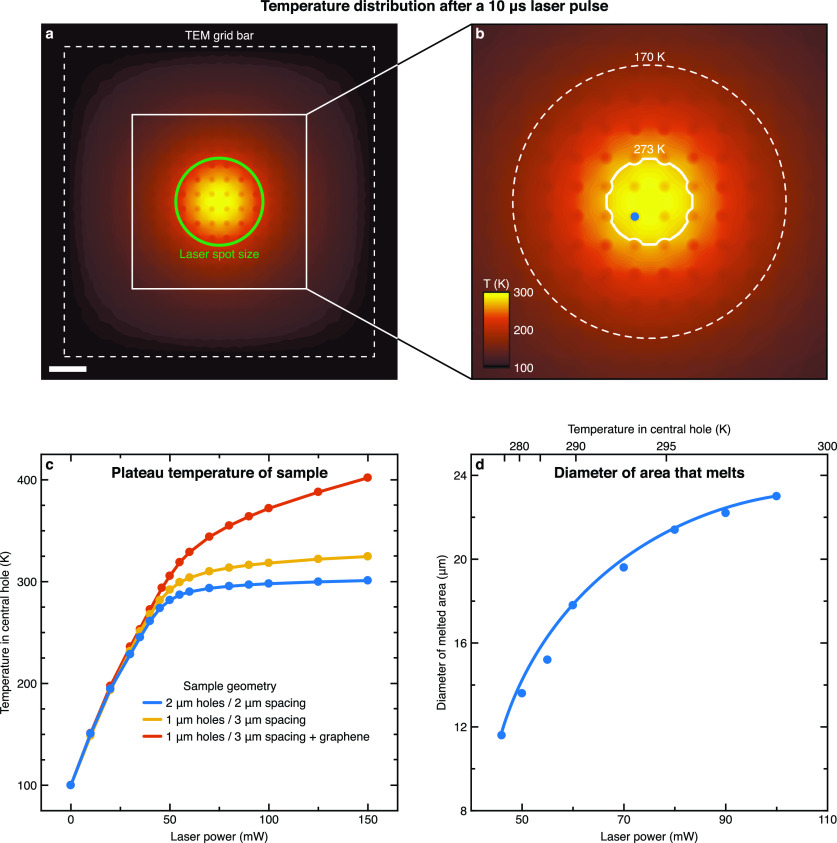
Heat transfer simulations of a melting and revitrification experiment. (a,b) Temperature distribution of a cryo sample after irradiation with a 10 *μ*s laser pulse (46 mW, 24 *μ*m spot size, indicated with a green circle). Scale bar, 10 *μ*m. The white line in (b) indicates the isotherm at 273 K, and the dashed circle represents the boundary of the crystalline region from the experiment in Fig. 3(b), at which the simulation predicts a temperature of 170 K. (c) Plateau temperature of the sample [at the position of the blue dot in (b)] as a function of laser power for different sample geometries. (d) Diameter of area that melts as a function of the laser power [probed at the position of the blue dot in (b)]. The curve serves as a guide for the eye.

Simulations of the temperature evolution of the sample in the experiment of Fig. 3 corroborate our interpretation of the observed phase behavior and provide further insight (Note S3 and Fig. S4). In agreement with the experiment, the temperature in the holes closest to the laser focus barely exceeds the melting point at the end of the 10 *μ*s laser pulse [46 mW, as in the experiment, see also Fig. S5(a)]. The corresponding temperature distribution of the cryo sample is depicted in Fig. 5(a), with the laser spot indicated by a green circle. While the sample in the holes closest to the laser focus has reached a temperature of 273 K, regions close to the bars of the specimen grid have remained at the initial temperature of 100 K. The regular pattern of holes in the gold film support manifests itself in lower sample temperatures at the sites of the holes. As we discuss in Fig. S5(b), adding a layer of graphene to the sample removes these local temperature gradients. An enlarged view of the area marked with a white square is shown in Fig. 5(b), with the 273 K isotherm highlighted in white. Notably, the size of the area enclosed by the isotherm closely matches that of the revitrified area in our experiment, suggesting that the revitrified area corresponds to the region that melts during the laser pulse. The dashed circle in Fig. 5(b) indicates the boundary of the crystalline region that we observe in the experiment. This boundary, for which the simulation predicts a temperature of about 170 K, is likely defined by the complex crystallization kinetics of the rapidly heated sample.

**FIG. 6.**
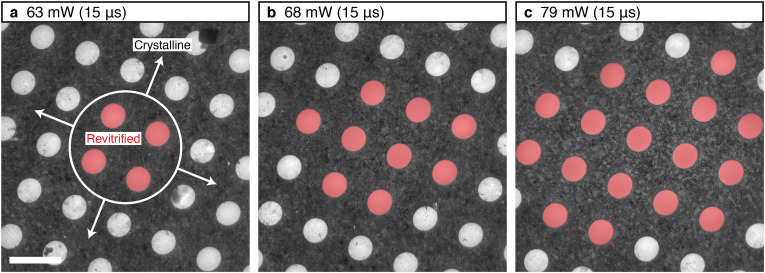
The size of the revitrified area increases with laser power. (a)–(c) Micrographs of cryo samples irradiated by a 15 *μ*s laser pulse of increasing power. Areas that melted and revitrified are highlighted in red. Scale bar, 4 *μ*m.

The temperature evolution of the sample and its phase behavior change in a characteristic fashion when the heating laser power is increased further. While the ice film in the holes closest to the laser focus [blue dot in Fig. 5(b)] heats up more rapidly [Fig. S5(c)], evaporative cooling limits the plateau temperature at which it stabilizes [blue curve in Fig. 5(c)]. Even at three times higher laser power, the temperature barely exceeds 300 K. This demonstrates that evaporative cooling can provide a negative feedback that stabilizes the plateau temperature for widely varying laser intensities, which facilitates control of the sample temperature. This negative feedback can be tuned by altering the sample geometry. For example, by reducing the hole size of the gold film from 2 to 1 *μ*m, the sample temperature increases by about 25 K at the highest laser powers (yellow curve). Much higher temperatures can be reached by adding a graphene layer between the holey gold film and the vitreous ice (red curve).^36^ This is desirable, for example, for the purpose of temperature jump experiments. With increasing laser power, the diameter of the area that is melted and revitrified increases, as shown in Fig. 5(d). The temperature reached in the holes closest to the laser focus increases from 273 to 298 K as the laser power is doubled. At the same time, the diameter of the revitrified area increases from 12 to 22 *μ*m. This is borne out experimentally in Fig. 6, which shows identical cryo samples after irradiation with 15 *μ*s laser pulses of increasing power. Here, the revitrified holes are highlighted in red. As the laser power is increased from 63 to 79 mW, the diameter of the revitrified area increases from 10 to 22 *μ*m.

## DISCUSSION AND OUTLOOK

Our experiments show that the phase behavior of the cryo sample provides a convenient means to infer details of its temperature evolution during a rapid melting and revitrification experiment. The laser power required for such an experiment can be calibrated *in situ* by determining the power needed to melt a devitrified area. By comparison with simulations, the power can then be increased, so as to heat the sample to a desired temperature above the melting point of ice. The success of an experiment can be immediately verified by the presence of a revitrified area surrounded by a crystalline region in which the sample has not melted. The diameter of the revitrified area is characteristic of the temperature distribution of the sample at the end of the laser pulse, which can be compared with simulations to reconstruct the entire temperature evolution of the sample. The diameter of the revitrified area can also be used to quickly assess *in situ* whether experiments on different areas of the grid have been carried out under identical conditions, allowing one to adjust the laser power on the fly if required. Our heat transfer simulations demonstrate that evaporative cooling provides a negative feedback that stabilizes the plateau temperature that the sample reaches. Small changes in the power or alignment of the laser, as well as slight variations of the heat transfer properties between different areas, will therefore only lead to minor errors in the temperature. By changing the sample geometry, evaporative cooling can be tuned either to limit the maximum temperature of the sample or enable temperature jump experiments. Intriguingly, our experiments suggest that crystallization occurs during laser heating as the sample traverses “no man's land”. Time-resolved experiments with microsecond electron pulses^37^ should allow us to verify this assumption by observing the phase behavior of water in real time.^38–41^ Such experiments also offer the opportunity to study the crystallization kinetics of supercooled water, which is a rich topic in its own right.^31–34^ We note that the transient formation of cubic ice during the laser melting process does not pose a problem for preserving the structure of the embedded proteins. In fact, it has recently been shown that high-resolution structures of particles can be obtained in devitrified samples.^42^

## Data Availability

The data that support the findings of this study are available from the corresponding author upon reasonable request.
